# New York Letter

**Published:** 1892-04

**Authors:** Wm. L. Russell

**Affiliations:** 151 East 50th Street; New York


### *CORRESPONDENCE.*

---

NEW YORK, March 15, 1892.

APPENDICITIS.

This subject has been referred to so frequently in these letters this winter that it might be considered hackneyed were it not for its great importance, and for the great struggle for a settled plan of treatment which is going forward. Thus far the surgeons have the best of it, and the physicians are now trying to adapt themselves to the circumstances as gracefully as possible. No surer proof of the triumph of the advocate of the early operation is needed than the fact that the physicians are now trying to steal some of the glory which attaches to the introduction of the plan of treatment. No one here has done so much as Dr. McBurney to lay down general principles for the management of these cases, or to convince the profession at large of the value of early diagnosis and early operation. To him then be the glory.

At the last meeting of the Academy, Dr. McBurney related the histories of several cases as they had come to him during two weeks in December, which illustrated the different clinical features presented by the disease. In the first case the onset was acute, there being sudden pain, followed by sensitiveness and rapid rise of temperature on the second day. An immediate operation was demanded in such a case. The second case gave a history of a more gradual advance of the disease, with at last almost disappearance of the symptoms. A "cure" might have resulted if the patient had remained in bed. As it was, however, a tumor formed, tenderness at a point two inches anterior to the anterior superior spine of the ilium (known here as the McBurney point) appeared, and an operation was performed. It was necessary to open the peritoneum, but a small opening was made in the abscess, and its contents expressed upon a sponge, so that soiling was avoided. In the third case, which presented a history somewhat similar to the last, there was some difficulty in finding the appendix. When this occurred it should be felt for with the finger, or the caput coli should be located as a landmark from which the appendix could be readily found. The fourth case was that of a stout man who presented no very acute symptoms, but in whom the operation disclosed a large gangrenous appendix distended with liquid fæces. Prompt operation alone would avert death in such a case. Recurrent attacks was the principal feature of the fifth case. "These cases," said Dr. McBurney, "were not well in the intervals, but had usually vague pains and constipation." One patient on whom he had operated had had twelve attacks in a year. In fifty cases operated on by him there had been but one death, while the mortality of the expectant plan of treatment was at least twenty per cent. Rectal examination was not so valuable as suggested by some, as only large abscesses could be detected in that way.

Dr. Delafield, speaking from the standpoint of a physician, said that there was room for honest difference of opinion in regard to the first three cases cited by Dr. McBurney, as they were likely to do well without any operation. The surgeon had the best of the argument, however, for unless operation was done the patient ran the risk of abscess and perforation. Besides, no recurrence was possible after operation; it might not occur without operation, but we could not be certain. The liability of yielding of the scar and the formation of hernia was the greatest objection to operation. There could be no difference of opinion about Dr. McBurney's fourth case. No such case could recover spontaneously. In fact, they were usually fatal. The whole appendix became gangrenous and septic poisoning supervened early, as in malignant diphtheria. There might be no pus, but unless operation was done before the septic poisoning appeared a fatal result was almost inevitable.

Dr. L. A. Stimson had seen three fatal cases within the past month in which a policy of waiting was pursued with the expectation of making a simple incision when the abscess, which all agreed was present, should point. Septic peritonitis resulted. He believed that the majority of medical cases which recovered had a recurrence. It was better to have a weak point in the abdominal wall than a soft spot in the appendix. Weakness of the abdominal wall was much more liable to follow when the operation was long delayed. Dr. Kinnicutt said that thirteen cases had been treated by him in the ten months last past. Of these nine were catarrhal, of which six were operated upon successfully, the remaining three recovering by resolution. In three others there was perforation and gangrene. Two recovered after operation, the third died of septic peritonitis. One case was tubercular. The frequency of recurrences of appendicitis had been noticed by him, and it appeared to him that such a result was more liable when a tumor remained after an attack, or a sense of local discomfort. In most catarrhal cases, a sense of discomfort often preceded the attack for a varying period. When perforation occurred, a sudden onset of pain and signs of shock was often followed by a misleading period of quiescence, which soon gave place to signs of a localized peritonitis, and rise of temperature. The initial treatment of all cases should not be by laxatives, but rather by sufficient opium to relieve pain and control peristalsis.

Dr. Abbe said that the claim that recurrent appendicitis did not suppurate was not well founded. The responsibility for the treatment of this disease now rested with the physicians; the surgeons had made up their minds. In many instances the surgeon was called too late. He had operated upon four cases successfully, in which general peritonitis was present. Draining by counter openings in the loins had been adopted in these cases.

Dr. Wm. H. Draper said that it was right that the responsibility should rest with the physician, who should call upon the surgeon, just as he used other therapeutic measures, when he saw that his services were required. A diagnosis was not always easy. In one instance stricture of the ileum was disclosed by the operation; and in another salpingitis with an external abscess. In doubtful cases, it was better to make an exploratory incision.

Dr. Loomis was of the opinion that as post mortems showed that appendicitis did sometimes recover, and that a recurrence did not follow, we could safely divide our cases into mild and severe, the general aspect of the patient, rather than the pulse and temperature, being followed as a guide. First attacks were usually mild, most severe cases giving a history of a previous attack. Therefore, if a previous attack could be excluded, and there were no severe symptoms, operation might be deferred. In cases beginning with very acute symptoms, an operation should be done without delay.

Dr. McBurney said in closing that by making the incision in the body of the muscle rather than near the margin of the rectus, the tendency to hernia would be lessened and perhaps avoided.

EMPYEMA.

An interesting discussion of this subject took place at the last meeting of the section on Pædiatrics. The question of diagnosis was presented by Dr. J. W. Brannan, who said that there were two constant and invariable diagnostic signs, displacement of the heart and dullness. These two signs were sufficient grounds for the use of the exploring needle, which alone was satisfactory. There was seldom much distension of the chest wall in children, owing to the feeble resistance to pressure offered by the lung. Auscultatory signs might also be misleading, as distinct respiratory sounds may be heard, though feeble.

The question of operation was discussed by Dr. J. H. Ripley. His practice was to operate in the simplest manner by making an incision in an intercostal space, and enlarging it with a blunt-pointed bistoury on a grooved director. The soft rubber drain age tube was then introduced, and allowed to remain there until the cavity began to fill when it was gradually removed. He did not think it necessary to remove a portion of rib, although this practice was advocated by some. He did not wash out the pleural cavity unless there was fetor, and then plain warm water was used. The operation should be performed as soon as the diagnosis was made, but a few days more or less was seldom important.

Dr. J. W. Roosevelt spoke about the causes of expansion of the lung after removal of the effusion. He said that compression of the lung seldom occurred, as its power of elastic recoil and the power of expansion of the chest wall would have first to be overcome. Much formation of granulation tissue on the pleural surfaces might delay or even prevent expansion, and eventually changes might occur in the elastic elements of the lung tissue. Experiments made a number of years ago by Dr. A. H. Smith showed that while the opening in the chest wall was large, the principal force in producing expansion of the lung was the air forced from the opposite lung during expiration ; as the opening grew smaller, however, expansion during inspiration began also. If the opening were stopped early, the air remaining would be absorbed. The contraction of granulation tissue between the pleural surfaces was another force operating to produce expansion. The operation should be done early and at the lowest point of the chest cavity. A chest full of air was better than a chest full of pus.

Dr. Caille said that he was convinced that primary empyema did sometimes occur, as he had seen a double empyema at an autopsy in a child whom he had examined a short time before and found perfectly well.

Dr. L. E. Holt had never seen a primary empyema. In cases occurring after scarlet fever, and thought to be primary, he had always found a pneumonia at the post mortem. Dr. Ripley said, in relation to diagnosis, that he regarded bronchial breathing over the retracted lung as a very valuable sign. Dr. Roosevelt said, however, that this could not be relied upon, for bronchial breathing and bronchial voice were sometimes heard over the whole chest. Dr. A. H. Smith considered ægophony as diagnostic when present.

Dr. R. H. M. Dawbarn said that in a case in which he had operated in an adult death by shock resulted from washing out the cavity. He could give no reason for this, as he thought every precaution had been taken.

Dr. J. E. Winters was of the opinion that undiagnosticated cases died either of exhaustion or of tuberculosis.

### HYPERTROPHIED TONSILS.

The question of when and how hypertrophied tonsils should be removed was considered at a recent meeting of the Academy.

Dr. M. J. Asch introduced the subject. He thought that tonsils should be removed whenver they embarrassed respiration. He thought excision dangerous, and preferred galvano-puncture, or removal by the galvanic loop.

Dr. F. H. Bosworth said that the existence of a tonsil was an indication for its removal. There was no tonsil apparent in a healthy throat. It was diseased lymphatic tissue which did not shrink and disappear at puberty as was sometimes claimed, but was usually a source of irritation in the pharynx. Whenever, then, there were any symptoms he advised removal. The best mode of removing was by means of the tonsillotome, and he regarded that of Mathieu as the best. The operation was not dangerous, and was very simple. It was better than galvano-puncture, which often produced considerable reaction, and required a number of sittings.

Dr. Bryson Delavan believed that the normal tonsil was very thin and concave. When it was large enough to excite observation it should be removed. When it was evidently diseased, or when it was the cause of recurrent attacks of quinsy or follicular tonsillitis, removal was also advisable. In children, and all under eighteen were children, excessive hemorrhage was very rare. In these then the tonsillotome should be used. When the hemorrhagic diathesis was present, or when a pulsating artery was visible, cutting was contraindicated. In adults the small sclerotic tonsil offered the greatest danger. He preferred Mackenzie's modification of Physick's tonsillotome. He did not care much for the cautery; it produced as great a reaction as the tonsillotome, and was tedious. He had seen five cases of diphtheria follow its use, and not one after the cutting operation.

Dr. Beverly Robinson did not think that every large tonsil should be removed, nor did he think it necessary to always remove the whole gland. He had found that the majority of tonsils could be sufficiently reduced in size by galvano-puncture, six sittings being as a rule all that were necessary.

Dr. Jacobi said that the mental hebetude which had been observed in those suffering from enlarged tonsils was not, as some supposed, due to the interference with respiration, but rather to the influence exerted on the lymphatics at the base of the brain.

WM. L. RUSSELL.

151 *East* 50*th Street.*

